# Errors in Table 1

**DOI:** 10.1001/jamanetworkopen.2021.45346

**Published:** 2022-01-04

In the Original Investigation titled “Identification and Validation of Plasma Metabolomic Signatures in Precancerous Gastric Lesions That Progress to Cancer,”^1^ published June 22, 2021, there were errors in a column of 95% CI values in Table 1. The odds ratios were correct, but each 95% CI given in the OR (95% CI) column under the IM/LGIN vs SG/CAG heading within the Validation set section was incorrect. This article has been corrected.^1^

## References

[1] Huang S, Guo Y, Li ZW, . Identification and validation of plasma metabolomic signatures in precancerous gastric lesions that progress to cancer. JAMA Netw Open. 2021;4(6):e2114186. doi:10.1001/jamanetworkopen.2021.14186 34156450 PMC8220475

